# A comprehensive profile of chemokines in the peripheral blood and vascular tissue of patients with Takayasu arteritis

**DOI:** 10.1186/s13075-022-02740-x

**Published:** 2022-02-16

**Authors:** Xiufang Kong, Sifan Wu, Xiaojuan Dai, Wensu Yu, Jinghua Wang, Ying Sun, Zongfei Ji, Lingying Ma, Xiaomin Dai, Huiyong Chen, Lili Ma, Lindi Jiang

**Affiliations:** 1 Department of Rheumatology, Zhongshan Hospital Affiliated to Fudan University, Shanghai, China; 2 Center of Clinical Epidemiology and Evidence-based Medicine, Fudan University, Shanghai, China

## Abstract

**Background:**

Takayasu arteritis (TAK) is a chronic granulomatous large vessel vasculitis with multiple immune cells involved. Chemokines play critical roles in recruitment and activation of immune cells. This study aimed to investigate chemokine profile in the peripheral blood and vascular tissue of patients with TAK.

**Methods:**

A total of 58 patients with TAK and 53 healthy controls were enrolled. Chemokine array assay was performed in five patients with TAK and three controls. Chemokines with higher levels were preliminarily validated in 20 patients and controls. The validated chemokines were further confirmed in another group of samples with 25 patients and 25 controls. Their expression and distribution were also examined in vascular tissue from 8 patients and 5 controls. Correlations between these chemokines and peripheral immune cells, cytokines, and disease activity parameters were analyzed. Their serum changes were also investigated in these 45 patients after glucocorticoids and immunosuppressive treatment.

**Results:**

Patients and controls were age and sex-matched. Twelve higher chemokines and 4 lower chemokines were found based on the chemokine array. After validation, increase of 5 chemokines were confirmed in patients with TAK, including CCL22, RANTES, CXCL16, CXCL11, and IL-16. Their expressions were also increased in vascular tissue of patients with TAK. In addition, levels of RANTES and IL-16 were positively correlated with peripheral CD3^+^CD4^+^ T cell numbers. Close localization of CCL22, CXCL11, or IL-16 with inflammatory cells was also observed in TAK vascular tissue. No correlations were found between these chemokines and cytokines (IL-6, IL-17, IFN-γ) or inflammatory parameters (ESR, CRP). No differences were observed regarding with these chemokines between active and inactive patients. After treatment, increase of CCL22 and decrease of RANTES and CXCL16 were found, while no changes were showed in levels of CXCL11 and IL-16.

**Conclusions:**

CCL22, RANTES, CXCL16, CXCL11, and IL-16 were identified as the major chemokines involved in the recruitment of immune cells in the vascular tissue of patients with TAK. Additionally, the persistently high levels of CCL22, CXCL11, and IL-16 observed after treatment indicate their role in vascular chronic inflammation or fibrosis and demonstrate the need for developing more efficacious treatment options.

**Supplementary Information:**

The online version contains supplementary material available at 10.1186/s13075-022-02740-x.

## Background

Takayasu arteritis (TAK) is a type of large vessel vasculitis that predominantly occurs in female patients of reproductive age (<40 years) and leads to vascular thickness, stenosis, and even occlusion of the aorta and its branches [1]. TAK is more prevalent in Asian countries, such as China, Japan, Korea, and Turkey, than in western countries [2].

TAK is characterized by chronic inflammation, and multiple immune cells participate in the development of TAK. High numbers of helper T cells, follicular helper T cells, CD8^+^T cells, CD14^+^ monocytes, and neutrophils cells have been found in the peripheral blood of patients with TAK [3]. In addition, various types of immune cells, such as macrophages, lymphocytes, and dendritic cells, have been observed in vascular lesions from patients with TAK [4]. Thus, the recruitment and activation of immune cells appear to be closely related with the development of vascular lesions in TAK.

Chemokines play critical roles in the recruitment of immune cells from the peripheral blood to injured tissue. For example, in giant cell arteritis (GCA), another large vessel vasculitis, CXCL9, CXCL10, and CXCL11 were reported to mediate macrophage infiltration in vascular lesions [5]. In TAK, vascular lesions originate from the vascular adventitia. The vascular adventitia is rich in vasa vasorum, which can transport peripheral immune cells to active vascular lesions in the early stage of lesion development [6]. In TAK patients, the levels of RANTES, CCL2, CCL20, CXCL8, and CXCL10 have been reported to be elevated in the peripheral blood, and their levels were correlated with disease activity [7, 8]. However, the profile of chemokines in TAK has not been clearly elucidated.

Apart from the role of chemokines in the chemotaxis of immune cells from the peripheral blood to injured lesions, chemokines may also participate in the migration of immune cells through vascular layers in TAK. TAK lesions develop from the outside to the inside of vascular tissue [9]. In our previous study, we found that macrophage distributed differently in different stages [10]. Additionally, the expression of CCL2, the main chemokine associated with macrophages, was consistent with the distribution of macrophages [10]. These findings indicate that chemokine also plays an important role in the migration of immune cells within vascular tissue in TAK. However, the expression of chemokines in vascular tissues in TAK is still unclear.

The aim of this study is to explore the chemokine spectrum in the peripheral blood as well as vascular tissue from patients with TAK and investigate its potential role in the pathogenesis of TAK.

## Methods

### Study population

A total of 58 naive patients with TAK and 53 healthy controls were enrolled in this study from Zhongshan Hospital, Fudan University, Shanghai, China, between January 1 and December 31, 2020. Patients were diagnosed according to the 1990 American College of Rheumatology TAK classification criteria [11]. The study design is presented in Supplementary Fig. 1. Among the study participants, 50 patients with TAK and 48 healthy controls were selected for serum examination, while the remaining 8 patients and 5 controls were selected for vascular tissue examination. The patients for vascular tissue examination were scheduled to undergo surgery and were treatment-naive at the time of surgery. Vascular control samples were obtained from apparently healthy donors for heart (ascending aorta) or liver (abdominal aorta) transplantation. The study protocol was approved by the Ethics Committees of Zhongshan Hospital (B2016-168) and conformed to the ethical guidelines of the 1975 Declaration of Helsinki. Written informed consent was obtained from all the participants in this study.

### Data collection and clinical assessment

Patient demographic and clinical data were recorded, including symptoms/signs, laboratory results, and imaging examination results. Laboratory parameters included inflammatory factors (erythrocyte sedimentation rate [ESR] and serum C-reactive protein [CRP]) and immune cell numbers or proportions in the peripheral blood. These immune cells were detected by routine flow cytometry, including monocytes (CD14^+^), lymphocytes (CD3^+^), CD4^+^ T cells (CD3^+^CD4^+^), CD8^+^ T cells (CD3^+^CD8^+^), B cells (CD19^+^), and natural killer (NK) cells (CD56^+^). A panel of fluorochrome-labeled monoclonal antibodies including CD3 (FITC, BioLegend), CD4 (PE-Cy7, BioLegend), CD8 (PE-Texas Red, BioLegend), CD14 (BV510, BioLegend), CD19 (PerCP/Cyanine5.5, BioLegend), and CD56 (APC, BioLegend) were used in flow cytometry and detected by BDFACSAriaIII. The gating strategy was shown in Supplementary Fig. 2. All the tests were conducted at the central laboratory of our center. Imaging examinations consisted of whole-body magnetic resonance angiography or computed tomographic angiography to assess vascular involvement.

Vascular types according to imaging findings were evaluated according to the imaging classification system created by Hata [12]. Disease activity was assessed based on the NIH activity criteria [13].

### Proteome profiler human chemokine array

Of the 50 patients and 48 healthy controls for serum examination, five patients with TAK and three age- and gender-matched healthy controls were chosen for the chemokine array assay (ARY017; R&D Systems Inc., Minneapolis). According to the instructions of the manufacturer, 31 chemokines were detected (Supplementary Fig. 3A). Results were expressed as the average signal (pixel density) of duplicate spots of each chemokine after the average background signal was subtracted. A 15% higher or lower difference in the average signal between patients and controls was indicative of a differentially expressed chemokine.

### Enzyme-linked immunosorbent assay

Based on the chemokine array results, 12 chemokines with higher levels in patients with TAK were preliminarily verified in 20 patients with TAK and 20 healthy controls by enzyme-linked immunosorbent assay (ELISA). These chemokines were CCL22, RANTES, CXCL16, CXCL11, IL-16, CCL1, CCL17, CCL19, CCL20, CXCL1, CX3CL1, and XCL1. After preliminary verification, five chemokines (CCL22, RANTES, CXCL16, CXCL11, and IL-16) with higher levels in TAK cases were further detected in the remaining 25 patients with TAK and 25 controls for further confirmation.

To clarify the effects of glucocorticoids and immunosuppressants on the expression of the chemokines, the serum levels of these five chemokines were also detected in the 45 patients with TAK after treatment. To investigate immune responses in the patients with TAK, the expression of relevant cytokines, including IL-6, IL-17, and IFN-γ was also detected in the 45 patients (baseline and post-treatment) and 45 controls. Commercial ELISA kits purchased from Boster Bio (CA, USA) were used to detect these chemokines and cytokines.

### Immunofluorescence and immunohistochemistry

To evaluate vascular infiltration upon vascular tissue, immunofluorescence for CD3 (ab16669, Abcam, MA, USA), CD19 (ab245235, Abcam, MA, USA), and CD68 (ab213363, Abcam, MA, USA) staining was performed. During this process, the deparaffination, rehydration, antigen retrieval, and blocking process were conducted as previously described [14]. During the staining process, CD68 antibody was firstly incubated at 4 °C overnight. On the 2nd day, after washing with PBS supplemented with 0.01% Tween-20, the corresponding secondary antibody was applied at room temperature for 1 h. Then, Tyramide-CY3 was used to detect the primary antibody binding. Then, the second antigen retrieval process was conducted for CD3 staining. The staining process were same to the first antigen and detected by Tyramide-FITC. After this, the slides went through antigen retrieval process again. CD19 was finally detected by CY5 conjugated secondary antibody. At last, the slides were mounted with anti-fluorescence quenching agent containing DAPI.

For the immunohistochemistry analysis, primary antibodies against CCL22 (abs118802; Absin, Shanghai, China), RANTES (abs131231; Absin, Shanghai, China), CXCL16 (abs122925; Absin, Shanghai, China), CXCL11 (ab9955; Abcam, MA, USA), and IL-16 (ab207181; Abcam, MA, USA), and their corresponding secondary antibodies were applied.

All the slides were digitally scanned using a 3DHISTECH scanning microscope, and images were viewed and selected using CaseViewer 2.4.0 (3DHISTECH Ltd., Hungary). In the quantification process, 10 views (about 0.15mm^2^) upon 400X magnification within the greatest inflammatory infiltrates were selected from each slide. The average number of CD3 or CD19 or CD68-positive cells were calculated using ImageJ and graded as +++ (the average number > 100/0.15mm^2^), ++ (the average number 50–100/0.15mm^2^), + (the average number 10–50/0.15mm^2^), or - (the average number <10/0.15mm^2^). Chemokine expressions were semi-quantified by IHC profiler in ImageJ. The results were presented as negative (-), weak positive (+), moderate positive (++), and strong positive (+++).

### Evaluation of the clinical significance of the identified chemokines

Distribution of the identified five chemokines (CCL22, RANTES, CXCL16, CXCL11, and IL-16) within inflammatory cells in vascular tissue and correlations between chemokine levels and immune cell (CD4^+^ T cell, CD8^+^ T cell, CD14^+^ monocyte, CD19^+^ B cell, and CD56^+^ NK cell) numbers in the peripheral blood were analyzed to investigate their potential role in immune cell recruitment. In addition, correlations between these chemokines and cytokines (IL-6, IL-17, and IFN-γ) were analyzed to explore their potential role in TAK immune response. Moreover, the levels of these five chemokines were compared between patients with active and inactive disease, and their correlations with inflammatory parameters (ESR and CRP) were examined to clarify their role in disease activity. Finally, changes in these chemokines were analyzed after glucocorticoids and immunosuppressive treatment.

### Statistical analysis

Measurement data, such as chemokine levels, ESR, and CRP, were expressed as mean ± standard deviation, while enumeration data, such as patients with active/inactive disease, were presented as frequencies and percentages. Student’s *t* test or paired *t* test were used to compare data that was normally distributed, while Mann-Whitney test or Wilcoxon rank test were used to compare non-parametric data. In the correlation analysis, Pearson correlation analysis was performed to analyze normal distribution data, while Spearman correlation analysis was applied for non-parametric data. All statistical analyses were performed using SPSS version 20.0 (Chicago, IL, USA). Graphs were generated using GraphPad Prism 5 (GraphPad Software Inc., USA). *P* values <0.05 were considered to indicate statistical significance.

## Results

### Patient characteristics

The patients with TAK and healthy controls selected for serum examination were matched for age (TAK: 34.44 ± 13.25 years, control: 37.85 ± 5.10 years, *p* = 0.27) and female to male ratio (TAK: 41:9, control: 36:12, *p* = 0.40). The clinical characteristics of patients with TAK included in the serum examination are listed in Table 1. Among the 50 patients with TAK, 40 (80%) had active disease at the time of enrollment. No significant differences were observed in the clinical characteristics (*p* > 0.05 for all parameters, Table 1) of patients (*n* = 5) selected for chemokine screening and those selected for chemokine validation (*n* = 45).

**Table 1:** Clinical characteristics of patients included in the serum examination

Clinical Parameters	Patients with TAK				Healthy controls (***n*** = 48)
	Total (***n*** = 50)	For chemokine screening (***n*** = 5)	For chemokine validation (***n*** = 45)	***p***	
**General information**					
Gender ratio (F:M)	41:9	5:0	36:9	057	36:12
Age at diagnosis (mean ± SD, y)	34.40 ± 13.25	28.80 ± 6.61	35.07 ± 13.69	0.32	/
Disease duration (months)	6.00 (2.00–15.00)	12.00 (1.50–30.0a0)	6.00 (2.00–12.00)	0.68	/
Active disease status (n, %)	40 (80.00)	3 (60)	37 (82.22)	0.26	/
**Clinical symptoms**					
Weakness (n, %)	11 (22.00)	2 (40)	9 (20.00)	0.30	/
Fever (n, %)	6 (12.00)	1 (20)	5 (11.11)	0.49	/
Hypertension (n, %)	14 (28.00)	1 (20)	13 (28.89)	1.00	/
Claudication (n, %)	2 (4.00)	0 (0)	2 (4.44)	1.00	/
Chest distress (n, %)	9 (18.00)	0 (0)	9 (20.00)	0.57	/
Dizziness (n, %)	24 (48.00)	3 (60)	21 (46.67)	0.66	/
Pulselessness/weak pulse (n, %)	17 (34.00)	2 (40)	15 (33.33)	1.00	/
Vascular bruits (n, %)	17 (34.00)	3 (60)	14 (31.11)	0.32	/
**Lab results**					
Hemoglobin (mean ± SD, g/L)	119.41 ± 15.20	115.00 ± 13.93	119.62 ± 15.35	0.50	132.69 ± 13.42
WBC (mean ± SD, 10^9^/L)	8.99 ± 5.87	9.51 ± 2.47	8.95 ± 6.10	0.97	6.23 ± 2.06
PLT (mean ± SD, 10^9^/L)	293.47 ± 93.70	346.25 ± 140.67	288.78 ± 89.18	0.12	258.46 ± 61.55
ESR (mean ± SD, mm/H)	44.18 ± 30.18	59.50 ± 31.69	42.82 ± 30.04	0.29	/
CRP (mean ± SD, mg/L)	24.40 ± 26.69	32.13 ± 26.18	23.71 ± 26.92	0.55	/
IL-6 (mean ± SD, pg/ml)	11.80 ± 10.26	12.54 ± 10.28	11.71 ± 10.38	0.87	/
TNF-α (mean ± SD, pg/ml)	8.94 ± 7.99	4.93 ± 0. 68	9.42 ± 8.33	0.29	/
**Imaging type**					
I	13 (26.00)	4 (80)	9 (20.00)	**0.028**	**/**
IIa	2 (4.00)	0 (0)	2 (4.44)		/
IIb	8 (16.00)	0 (0)	8 (17.78)		/
III	1 (2.00)	0 (0)	1 (2.22)		/
IV	5 (10.00)	1 (20)	4 (8.89)		/
V	22 (48.89)	0 (0)	22 (46.67)		/

*SD* Standard deviation, *WBC* White blood cell, *PLT* Platelet, *ESR* Erythrocyte sedimentation rate, *CRP* C-reactive protein

The clinical characteristics of patients (*n* = 8) and controls (*n* = 5) selected for vascular tissue examination are listed in Supplementary Table 1. The main reasons for surgery were aortic regurgitation (*n* = 7) and renal artery occlusion (*n* = 1). Thus, ascending aortic specimens (*n* = 7) and a renal artery specimen (*n* = 1) were obtained. In addition, apparently normal specimens (control) of the ascending aorta (*n* = 3) and abdominal aorta (*n* = 2) were obtained from donors for heart transplantation (*n* = 3) and liver transplantation (*n* = 2).

### Increased expression of CCL22, RANTES, CXCL16, CXCL11, and IL-16 in the peripheral blood of patients with TAK

The main functions of 31 chemokines in the array are listed in Supplementary Table 2. A representative image of the chemokine array results is shown in Supplementary Fig. 3A, and the combined results are shown in Supplementary Fig. 3B. Among the 31 chemokines, the average signal of 12 chemokines was over 15% higher in patients with TAK than in the healthy controls (Supplementary Fig. 3C a–l): CCL22, RANTES, CXCL16, CXCL11, IL-16, CCL1, XCL1, CX3CL1, CXCL1, CCL17, CCL19, and CCL20. In contrast, the average signal of four chemokines (CXCL10, CXCL7, CCL18, and CXCL4) was 15% lower in patients with TAK than in the healthy controls (Supplementary Fig. 3C m–p).

**Fig. 1:**
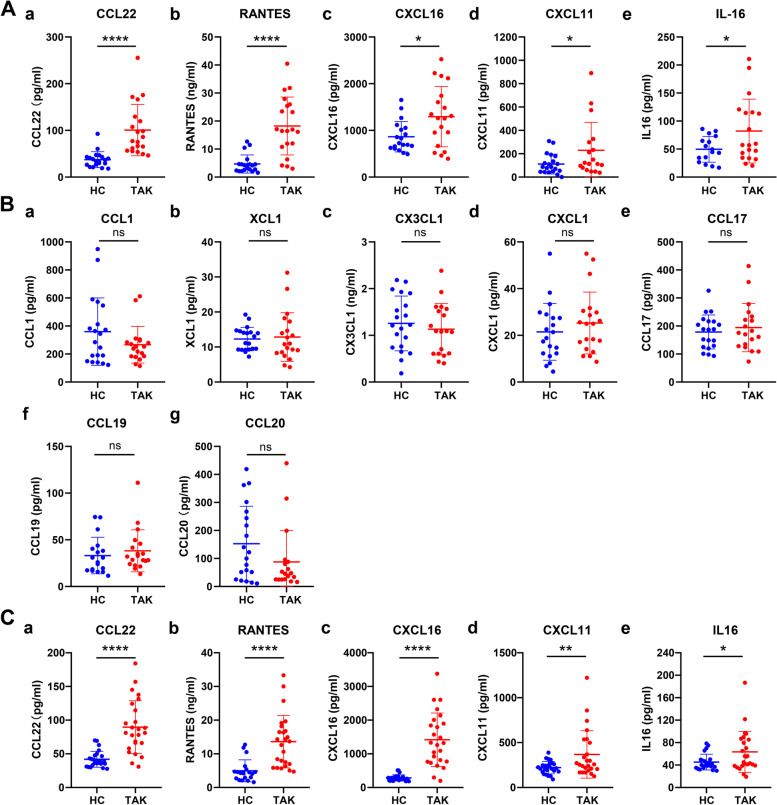
Validation of chemokine expression in the peripheral blood of patients with TAK. **A** Higher levels of CCL22 (a), RANTES (b), CXCL16 (c), CXCL11 (d), and IL-16 (e) were observed in TAK patients in contrast to the healthy controls (*n* = 20 in each group). **B** No differences were found in CCL1 (a), XCL1 (b), CX3CL1 (c), CXCL1 (d), CCL17 (e), CCL19 (f), and CCL20 (g) levels between patients with TAK and the controls (*n* = 20 in each group). **C** Higher levels of CCL22 (a), RANTES (b), CXCL16 (c), CXCL11 (d), IL-16 (e), IL-6 (f), and IL-17 (g) were confirmed in patients with TAK than in the controls (*n* = 25 in each group). *p < 0.05, ****p < 0.0001, ns = not significant

The 12 chemokines with significantly higher expression in patients with TAK were priliminarlily validated in 20 patients with TAK and 20 healthy controls. The results showed that the levels of five chemokines were significantly higher in patients with TAK than in the healthy controls (CCL22: *p* < 0.0001, RANTES: *p* < 0.0001, CXCL16: *p* = 0.01, CXCL11: *p* = 0.04, IL-16: *p* = 0.04) (Fig. 1A). However, no significant differences were found in the other seven chemokines (CCL1, XCL1, CX3CL1, CXCL1, CCL17, CCL19, and CCL20: *p* > 0.05 for all) (Fig. 1B).

To confirm these five chemokines with higher levels in patients with TAK, their levels were further detected and analyzed in another 25 controls and patients with TAK. The results also confirmed that these five chemokines were significantly higher in the patients with TAK (CCL22: *p* < 0.0001, RANTES: *p* < 0.0001, CXCL16: *p* < 0.0001, CXCL11: *p* < 0.01, IL-16: *p* = 0.03) (Fig. 1C). The data indicate that these five chemokines, CCL22, RANTES, CXCL16, CXCL11, and IL-16, might play a role in TAK development.

### Increased expression of CCL22, RANTES, CXCL16, CXCL11, and IL-16 in vascular tissue and their relationships with vascular and peripheral immune cell numbers in patients with TAK

**Fig. 2:**
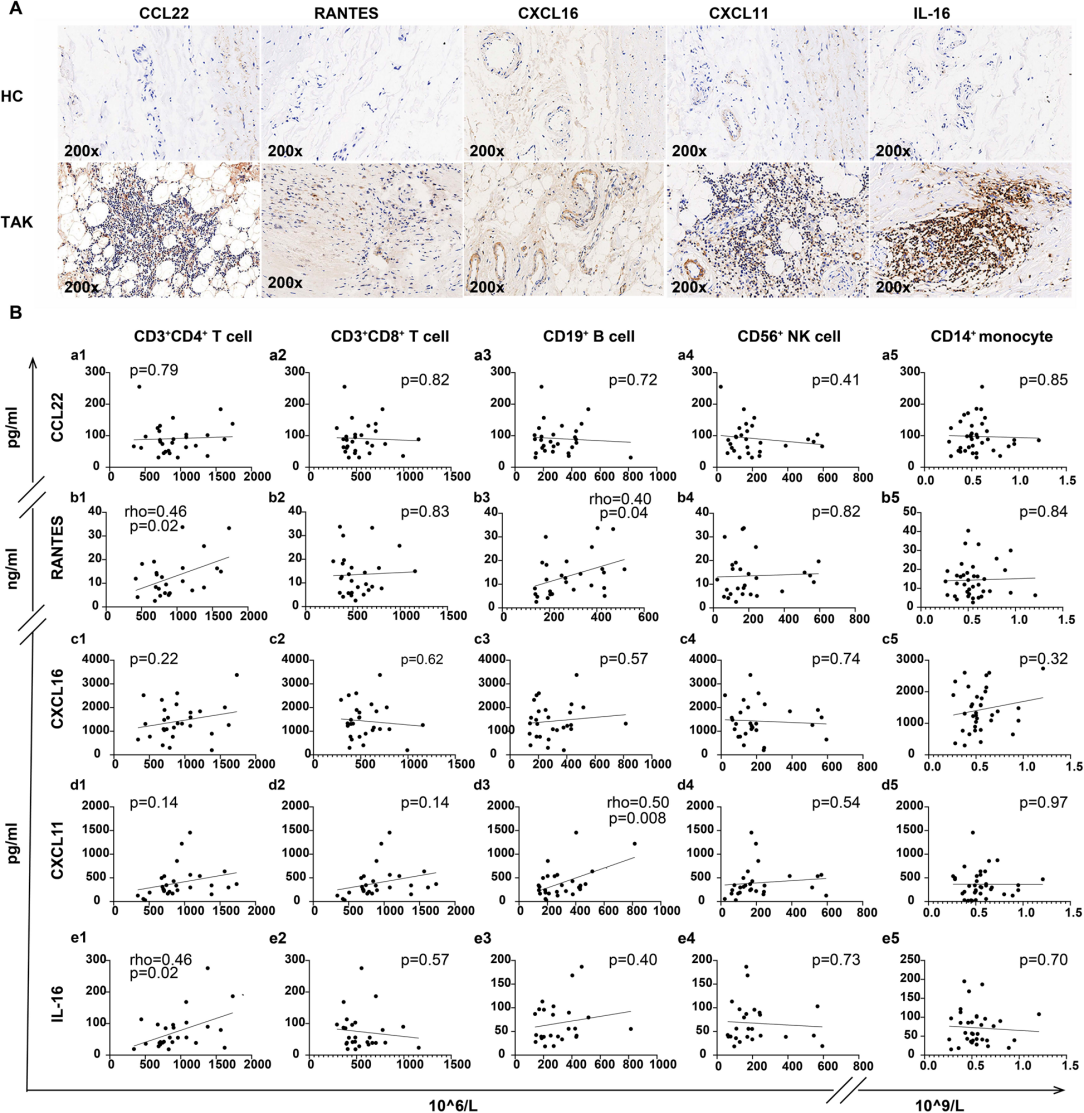
Relationship between increased chemokine levels and vascular or peripheral immune cell numbers in patients with TAK. **A** Representative immunohistochemical staining images of vascular expression of CCL22, RANTES, CXCL16, CXCL11, and IL-16 in patients with TAK and healthy controls. **B** Correlations between CCL22 (a1–a5), RANTES (b1–b5), CXCL16 (c1–c5), CXCL11 (d1–d5), and IL-16 (e1–e5) levels and CD3^+^CD4^+^ T cells, CD3^+^CD8^+^ T cells, CD19^+^ B cells, CD56^+^ NK cells, and CD14^+^ monocytes

The immunofluorescence staining showed different grades of inflammatory infiltrates (CD3^+^ or CD19^+^ or CD68^+^ cells) in vascular adventitia (Supplementary Table 1 and Supplementary Fig. 4). The expressions of these five chemokines were also increased in TAK vascular lesions (Supplementary Table 1 and Fig. 2A). Specifically, CCL22 was expressed in a weak positive to moderate positive grade (+ ~ ++) and mainly detected at sites with inflammatory cell infiltration (Fig. 2A: CCL22); RANTES was weak positively (+) expressed in vascular adventitia, which was not necessarily related to inflammatory cells (Fig. 2A: RANTES); CXCL16 was weak to strong positively (+ ~ +++) expressed and predominantly detected in microvessel walls in the adventitia (Fig. 2A: CXCL16); CXCL11 was moderate to strong positively expressed (++ ~ +++) in microvessel walls as well as lesions infiltrated with inflammatory cells (Fig. 2A: CXCL11); and IL-16 expression was also in a moderate to strong positive grade (++ ~ +++) and closely related with the distribution of inflammatory cells (Fig. 2A: IL-16).

Based on the observations above, the correlations between increased chemokine levels and peripheral immune cells were further analyzed among 45 patients (Fig. 2B). Compared with the findings in TAK tissue, the relationship between the chemokines and peripheral immune cells (CD3^+^CD4^+^ T cells, CD3^+^CD8^+^ T cells, CD19^+^ B cells, CD56^+^ NK cells, and CD14^+^ monocytes) exhibited a different pattern. Surprisingly, no correlations were found between CCL22 and immune cell numbers in peripheral blood (Fig. 2B a1–a5). Similarly, no correlations were observed between CXCL16 and immune cell numbers either (Fig. 2B c1–c5). However, RANTES was weakly correlated with CD3^+^CD4^+^ T cell number (rho = 0.46, *p* = 0.02, Fig. 2B b1) and CD19^+^ B cell number (rho = 0.40, *p* = 0.04, Fig. 2B b3), but it was not correlated with the other cell types (Fig. 2B b2, b4, b5). With regard to CXCL11, it was weakly correlated with CD19^+^ cell number (rho = 0.50, *p* = 0.008, Fig. 2B d3), but not with the number of the other immune cells (Fig. 2B d1, d2, d4, & d5). Interestingly, IL-16 was weakly correlated with CD3^+^CD4^+^ T cell number (rho = 0.46, *p* = 0.02, Fig. 2B e1), but not with the number of other types of cells (Fig. 2B e2–e5).

These results indicate that while the chemokine expression was localized with immune cell numbers in vascular tissue, the correlations of these chemokines with immune cell numbers in the peripheral blood were limited.

### Correlation between increased chemokine levels and inflammatory cytokine levels in the peripheral blood of patients with TAK

Higher levels of IL-6 (*p* < 0.001) and IL-17 (*p* = 0.004) in patients with TAK in contrast to the healthy controls (Supplementary Fig. 5A & B). However, IFN-γ levels were lower in the patients than in the healthy controls (*p* < 0.001, Supplementary Fig. 5C). These data indicate that higher chemokine levels were accompanied with increase in the levels of IL-6 and IL-17, which are the two main inflammatory cytokines involved in the development of TAK. The correlation analysis between these chemokines and cytokines (Supplementary Fig. 5D) indicated a weak correlation between RANTES and IL-6 levels (rho = 0.41, *p* = 0.02, Supplementary Fig. 5D b1). However, no correlations were found between the other chemokines and IL-6 or IL-17 levels (Supplementary Fig. 5D).

### Correlations between chemokine levels and disease status

Unfortunately, no differences were found in the levels of five chemokines between active and inactive patients (Supplementary Fig. 6A a–e), and no correlations were observed between these chemokines and the CRP (Supplementary Fig. 6B) or ESR (Supplementary Fig. 6C) levels either.

### Correlations between individual chemokine levels

The results showed that levels of CCL22, CXCL16, and IL-16 were mutually correlated. In particular, CCL22 was positively correlated with CXCL16 (*r* = 0.57, *p* < 0.0001, Supplementary Fig. 7A) and IL-16 (rho = 0.57, *p* < 0.0001, Supplementary Fig. 7B), and IL-16 was also positively related with CXCL16 (rho = 0.40, *p* = 0.005, Supplementary Fig. 7C).

### Effect of immunosuppressive treatment on the identified chemokines and TAK-related cytokines

The treatment included glucocorticoids combined with leflunomide (20 patients, 44.44%), cyclophosphamide (15 patients, 33.33%), tocilizumab (8 patients, 17.78%), or methotrexate (2 patients, 4.44%). The median treatment duration was 6 (interquartile range: 5–6) months. After treatment, 14 (31.11%) patients had active disease according to their NIH score.

**Fig. 3:**
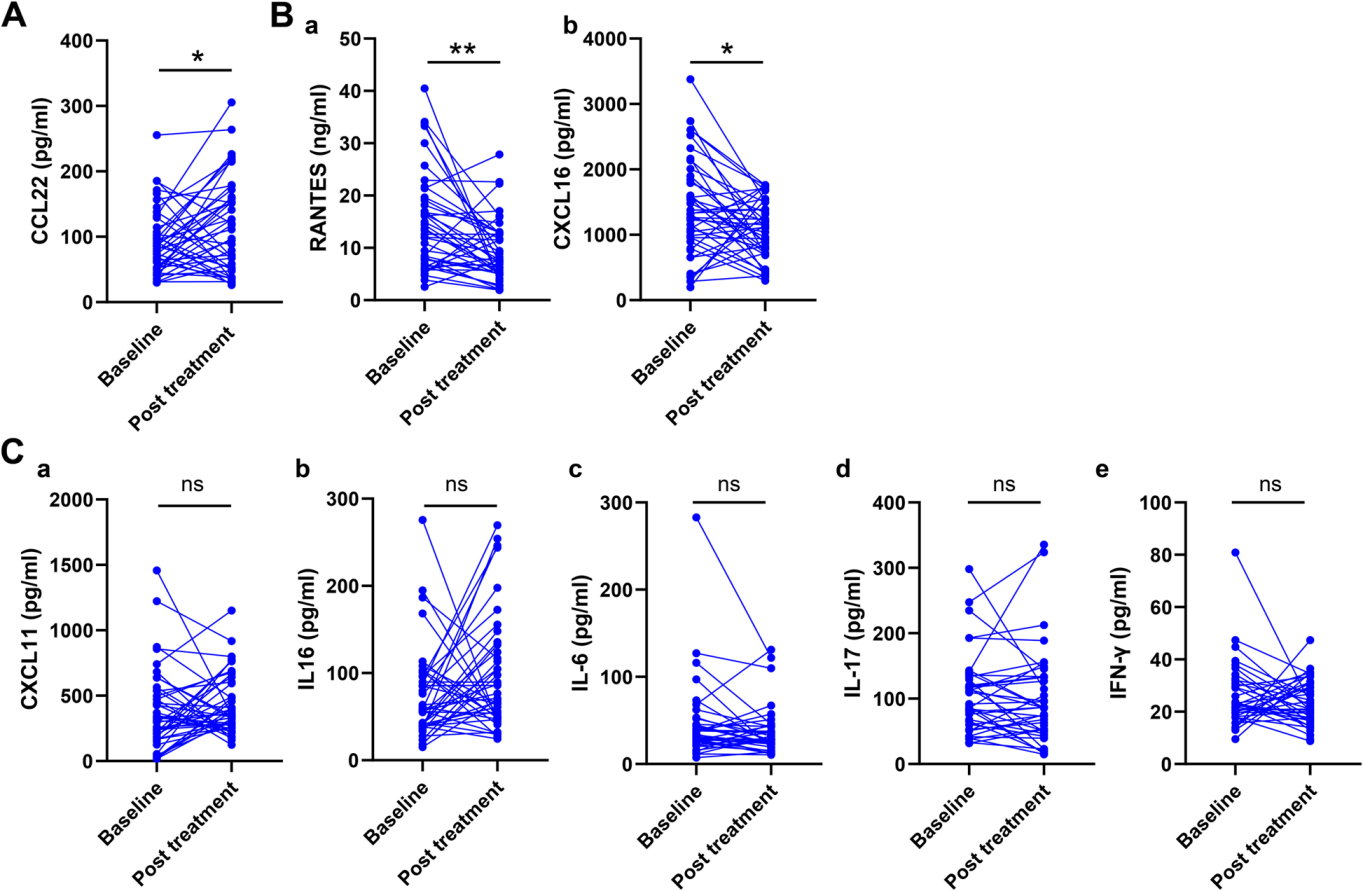
Changes in peripheral chemokine and cytokine levels after treatment in patients with TAK. **A** CCL22 levels were increased after treatment. **B** RANTES (a) and CXCL16 (b) levels were decreased after treatment. **C** No differences were observed in CXCL11 (a), IL-16 (b), IL-6 (c), IL-17 (d), and IFN-γ levels before and after treatment (*n* = 45 in each group).*p < 0.05, **p < 0.01, ns = no significance

After treatment, a further increase was observed in CCL22 levels (85.66 (52.39, 114.64) vs. 111.20 (51.71, 160.67), *p* = 0.03, Fig. 3A), while there was a significant decrease in RANTES (14.68±9.35 vs. 9.43±5.90, *p* = 0.001) and CXCL16 (1381.17±743.26 vs. 1083.03±447.39, *p* = 0.02) (Fig. 3B). However, no differences were observed in the levels of CXCL11 (328.35 (168.55, 535.43) vs. 352.13 (271.29, 604.85), *p* = 0.31, Fig. 3C a) and IL-16 (56.95 (39.24, 96.19) vs. 70.25 (48.60, 126.25), *p* = 0.11, Fig. 3C b). No changes were found in the levels of the three TAK-related cytokines (IL-6: 32.57 (25.46, 46.73) vs. 27.58 (22.34, 42.58), *p* = 0.08, IL-17: 86.97 (58.30, 130.54) vs. 74.01 (45.00, 126.88), *p* = 0.48, IFN-γ: 22.56 (18.10, 30.30) vs. 20.88 (16.60, 29.77), *p* = 0.39, Fig. 3C c–e) after treatment.

We found that changes in the examined chemokines after treatment differed across individual patients. Therefore, immune cell numbers, ESR, CRP, and cytokines changes in the peripheral blood after treatment were compared between patients with increased and decreased levels of each chemokine. However, no differences were observed in these indicators between the two groups (data not shown). Correlations between the changes in these chemokines were also analyzed, and the results indicated similar trends in the changes in CCL22, CXCL16, and IL-16 levels after treatment (Supplementary Fig. 7D–F).

**Fig. 4:**
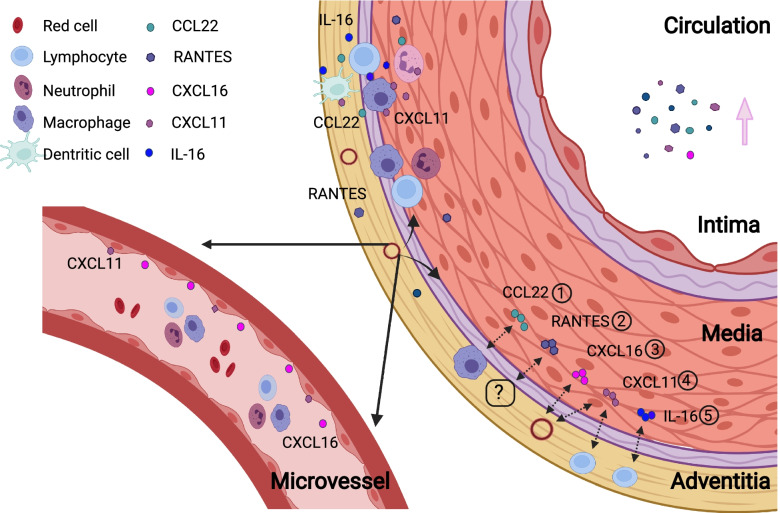
A proposed model illustrating chemokines discovered in the present study and their potential role in the pathogenesis of TAK. CCL22, RANTES, CXCL16, CXCL11, and IL-16 were increased in the peripheral blood as well as vascular tissue of TAK. In active vascular lesions, the infiltration was predominated by CD3^+^ T cells, a less proportion of CD68^+^ macrophage and CD19^+^ B cells. Among these five chemokines, CCL22, IL-16, and CXCL11 were distributed in vascular infiltration. RANTES was expressed in a relative low level. In addition, CXCL11 was also expressed in adventitial microvessels, while CXCL16 was mainly expressed in adventitial microvessel wall. Based on their functions, their potential roles in TAK were presumed as followed: ① CCL22 may participate in macrophage recruitment; ② RANTES is able to recruit multiple cells, but the specific cell type it functions needs further exploration; ③ CXCL16 probably involves in migration of peripheral immune cells from adventitial microvessels to vascular lesions; ④ CXCL11 may recruit active T cells as well as participate in the recruitment immune cells from microvessels; ⑤ IL-16 probably promotes CD4^+^ T cells infiltration

A summary of the characteristics of these five chemokines in patients with TAK is shown in Table 2. A proposed model illustrating chemokines discovered in the present study and their potential role in the pathogenesis of TAK was shown in Fig. 4.

**Table 2:** Summary of the chemokine profile in patients with TAK

Chemokines	CCL22	RANTES	CXCL16	CXCL11	IL-16
**Peripheral blood**					
Before treatment	↑	↑	↑	↑	↑
After treatment	↑↑	↓	↓	→	**→**
Positive correlation	CXCL16, IL16	CD3^+^CD4+ T cell number, CD19^+^ B cell number, IL-6 level	CCL22, IL-16	CD19^+^ cell number	CD3^+^CD4^+^ T cell number, CCL22, CXCL16
**Vascular tissue**					
Expression	+ to ++	+ to ++	+ to +++	++ to +++	++ to +++
Main Distribution	Area with inflammatory cell infiltration, mainly in the adventitia	Mainly in adventitia, scatteredly distributed, not necessarily correlated with inflammatory cell infiltration	Microvessel wall in the adventitia	Microvessel wall and area with inflammatory cell infiltration in the adventitia	Area with inflammatory cell infiltration, mainly in the adventitia
**Expression cell types**	Mainly macrophages, DCs, NK cells [15]	Macrophages, T lymphocytes, endothelial cells, platelets, synovial fibroblasts, etc. [16]	Macrophages, DCs, T cells, cytokine-stimulated SMCs and ECs, etc. [17]	Monocytes, endothelial cells, fibroblasts, etc.	Various cells, such as CD4^+^ T cells, CD8^+^ T cells, monocytes, and fibroblasts [18]
**Recruitment cell types**	Monocytes, dendritic cells, natural killer cells and for chronically activated T lymphocytes [19]	T cells, macrophages, NK cell, eosinophils, basophils, etc. [16]	T cell, NK cell	Activated T-cells	CD4^+^ lymphocytes, monocytes, and eosinophils [18]
**Role in biological processes**	Immune cell chemotaxis, Th2 response, allergy, fibrosis [19]	Immune cell chemotaxis, pro-inflammation pathways, angiogenesis [16, 20]	Immune cell chemotaxis, cell adhesion, role as a cell surface scavenger receptor [21]	Immune cell chemotaxis, Th1 response, angiogenesis [22]	Immune cell chemotaxis, T cell activation [23]
**Role in vasculitis/vascular disorders**	Increased expression in active EGPA, association with blood eosinophilia [24]	Increased expression in TAK and GCA, association with perivascular inflammation and immune cell infiltration [25, 26]	Regulation of inflammation in cardiovascular disease [27], promotion of angiogenesis in RA [28], role in atherosclerosis as a scavenger receptor for OxLDL [17]	Induction of macrophage infiltration in GCA [5], association with IFN-γ in mixed cryoglobulinemia and chronic hepatitis C infection with active vasculitis [29]	Association with vascular damage index in AAV, possible involvement in progressive fibrosis [30], role in the migration and invasion of VSMCs [31]
**Role in other disorders**	Regulation of EAE via macrophage accumulation [32], role in allergic diseases such as asthma and allergic rhinitis [33], role in pulmonary fibrosis [34]	Role in multiple tumors, promotion of carcinogenesis and invasiveness of tumor cells [35], involvement in antiviral response in viral infections [36]	Promotion of tumor cell proliferation, migration, invasion, and metastasis [21]	Role in multiple autoimmune disorders, such as autoimmune thyroiditis and autoimmune encephalomyelitis [37, 38], and promotion of tumor growth and metastasis [39]	Association with disease activity in SLE [40] and RA [41, 42]

↑ increase, ↓ decrease, ↑↑ further increase, → no changes, / not applicable or negative, +++ strongly positive, ++ moderately positive, + weakly positive

*MDC* Macrophage-derived chemokine, *DC* Dendritic cell, *NK* Natural killer, *EGPA* Eosinophilic granulomatosis with polyangiitis, *EAE* Experimental autoimmune encephalomyelitis, *TAK* Takayasu arteritis, *GCA* Giant cell arteritis, *SR-PSOX* Scavenger receptor for phosphatidylserine and oxidized lipoprotein, *RA* Rheumatoid arthritis, *I-TAC* Interferon-inducible T cell, a chemoattractant, *OxLDL* Oxidized low-density lipoprotein, *LCF* Lymphocyte chemoattractant factor, *AAV* Anti-neutrophil cytoplasmic antibody (ANCA)-associated vasculitis, *VSMCs* Vascular smooth muscle cells, *ECs* Endothelial cells, *SLE* Systemic lupus erythematosus

## Discussion

The present study demonstrates that the chemokines CCL22, RANTES, CXCL16, CXCL11, and IL-16 are elevated in the peripheral blood as well as vascular tissue of patients with TAK, and expression of CCL22, CXCL11, and IL-16 are distributed in vascular inflammatory lesions. These phenomena indicated that they may play a role in the recruitment of immune cells in vascular lesions of TAK. Further, after routine glucocorticoids and immunosuppressant treatment, RANTES and CXCL16 are decreased, while CCL22, CXCL11, and IL-16 present a persistent high levels. Thus, CCL22, CXCL11, and IL-16 may participate in the vascular chronic inflammation or fibrotic process of TAK.

The key functions of the five TAK-related chemokines, namely, CCL22, RANTES, CXCL16, CXCL11, and IL-16, identified in this study are reviewed in Table 2. CCL22 is mainly produced by macrophages, dendritic cells, and can recruit macrophages via CC chemokine receptor 4 [15, 19]. It has been implicated in inflammatory, allergic, as well as fibrotic disorders [24, 32–34]. In TAK, macrophage was a major cell type in vascular lesions [10]. Thus, CCL22 may be mainly derived from macrophages and involved in macrophage recruitment. CCL22 is also a biomarker of M2 macrophages [43]. It has been reported that M2 macrophages dominated M1 macrophages in TAK vascular lesions [44]. Our previous study also found macrophages experienced a M1 to M2 shift from untreated to treated vascular lesions [10]. This probably was an important reason for persistent high serum CCL22 levels in TAK. However, the relationship between CCL22 and vascular macrophages needs further verification.

RANTES is an inflammatory chemokine that exhibits chemotaxis towards multiple inflammatory cells, especially T cells via CCR5 [16, 20]. RANTES has been reported to play a role in multiple vascular diseases, tumors, and viral infections [25, 26, 35, 36]. In the present study, the increase of serum RANTES is consistent with previous findings [7, 25]. However, its expression in vascular tissue has not ever been studied. In GCA, RANTES was co-localized with leucocytes in vascular adventitia [26], but this phenomenon was not prominent TAK vascular tissue according to the results in the present study. In the peripheral blood, a correlation was observed between RANTES and IL-6 in this study, which indirectly suggested its participation in the inflammatory process of TAK. Finally, the decrease in RANTES levels after treatment indicated a response to glucocorticoids and immunosuppressants treatment.

CXCL16 participates in various pathological processes such as immune cell recruitment, cell interactions, angiogenesis etc. [17, 21], leading to its involvement in multiple disorders [27, 28]. Importantly, CXCL16 has been reported to mediate the adhesion of platelets or leucocytes to endothelial cells [45]. In the present study, CXCL16 was mainly expressed in the adventitial microvessel wall. Therefore, CXCL16 probably plays a critical role in recruitment of immune cell from the peripheral blood to vascular lesions via adventitial microvessels. In addition, CXCL16 has been reported to play a pro-angiogenic role by recruiting endothelial progenitor cells [28]. Angiogenesis is also increased in vascular lesions in TAK, but whether CXCL16 also participates in neovascularization in TAK remains further investigation. Similar to RANTES, CXCL16 also can be downregulated in response to glucocorticoids and immunosuppressive treatment.

CXCL11 acts as a chemokine for T cells and macrophages via binding to the receptor CXCR3 [22]. The CXCL11/CXCR3 axis has been implicated in multiple autoimmune disorders and tumors [5, 22, 29, 37–39]. In GCA, CXCL11 expression was also observed in vascular lesions, and blocking IFN-γ was found to reduce CXCL11 expression and further decrease macrophage infiltration [5]. In the present study, CXCL11 expression was mainly observed in microvessel wall and inflammatory lesions. This indicates its role in immune cell recruitment and activation in TAK. In the present study, persistently high levels of CXCL11 were found in the peripheral blood after glucocorticoids and immunosuppressive treatment, which may contributed to vascular chronic inflammation in TAK. Thus, it is worthy to explore whether CXCL11/CXCR3 axis also involved in the pathogenesis of TAK in future studies.

IL-16 has chemotactic function in immune cells, especially CD4^+^ T cells [18, 23]. Its expression was associated with tissue CD4^+^ T lymphocyte infiltration in multiple disorders [46–48]. In antineutrophil cytoplasmic antibody-associated vasculitis, IL-16 was correlated with the vasculitis damage index [30] and was presumed to be involved in vascular fibrosis. In addition, it was reported to participate in the migration and invasion of vascular smooth muscle cells (VSMCs) [31]. In the present study, the positive correlation between IL-16 levels and CD4^+^ T cell number and the close distribution of IL-16 to lymphocyte infiltrates also indicate an important role for IL-16 in CD4^+^ T cell recruitment. Further, a persistent high levels of IL-16 were observed in TAK after glucocorticoids and immunosuppressive treatment. Whether high levels of IL-16 contribute to chronic fibrosis in vascular lesions needs further exploration.

In the present study, although no correlations were observed between these chemokine levels and the disease activity markers ESR or CRP, high levels of IL-6 and IL-17 were detected along with the high levels of chemokines. This finding, combined with the close distribution of chemokines with vascular immune cells, implies that these chemokines may also be indicators of active immune responses in TAK. Additionally, the persistently high levels of CXCL11 and IL-16 after treatment probably indicates uncontrolled immune activation or their role in chronic vascular fibrosis. The correlations observed between CCL22, CXCL16, and IL-16 indicate that these chemokines may function in a synergistic way.

This study comprehensively investigated the chemokine profile of TAK and preliminarily explored the potential role of five identified chemokines in vascular immune cell recruitment. They may play different roles in vascular lesions by attracting different cell types. These findings provide important evidence for the pathogenesis of TAK, but there are also some limitations to this study. Firstly, in this study, four chemokines with lower levels screened from array had not been further validated. Lower levels of chemokines may reflect the usage of them by immune cells. Thus, it was worthy to have more research on them in future TAK studies. In addition, only five chemokines validated in the serum were detected in vascular tissue. Thus, certain critical chemokines in vascular tissue could have been overlooked due to inconsistencies between the peripheral blood and vascular tissue findings. Moreover, the treatment period was relatively short, so the effects of glucocorticoids and immunosuppressive agents on these chemokines need further verification Finally, the origins and roles of these chemokines remained to be explored further.

## Conclusions

CCL22, RANTES, CXCL16, CXCL11, and IL-16 are identified as the major chemokines that may involve in the recruitment of immune cells in the vascular tissue of patients with TAK. Additionally, the persistently high levels of CCL22, CXCL11, and IL-16 observed after treatment might suggest their participation in vascular chronic inflammation or fibrosis and demonstrate the need for developing more efficacious treatment options.

## Supplementary Information

**Additional file 1: Supplementary Figure 1.** Flow chart depicting the study design. **Supplementary Figure 2.** Gating strategies in flow cytometry for peripheral immune subsets analysis. **Supplementary Figure 3.** Results of chemokine array in the present study. **Supplementary Figure 4.** Vascular infiltration in patients with TAK. **Supplementary Figure 5.** Peripheral cytokine levels and their correlation with chemokine levels in patients with TAK. **Supplementary Figure 6.** Correlations between chemokines and disease activity markers in patients with TAK. **Supplementary Figure 7.** Correlations among different chemokines and changes in their levels after treatment. **Supplementary Table 1.** Clinical characteristics of the patients included for vascular tissue examination. **Supplementary Table 2.** Key functions of the five major chemokines.

## Data Availability

The datasets used and/or analyzed during the current study are available from the corresponding author on reasonable request.

## Abbreviations

TAK: Takayasu arteritis
GCA: Giant cell arteritis
ESR: Erythrocyte sedimentation rate
CRP: C-reactive protein
NK cells: Natural killer cells
ELISA: Enzyme-linked immunosorbent assay
VSMCs: Vascular smooth muscle cells
