# Supplementary material for: A comprehensive profile of chemokines in the peripheral blood and vascular tissue of patients with Takayasu arteritis
Source: Arthritis Res Ther. 2022 Feb 16;24:49. doi: 10.1186/s13075-022-02740-x (PMC8848964; doi:10.1186/s13075-022-02740-x)
Supplement: Supplementary file 1 — Additional file 1: Supplementary Figure 1. Flow chart depicting the study design. Supplementary Figure 2. Gating strategies in flow cytometry for peripheral immune subsets analysis. Supplementary Figure 3. Results of chemokine array in the present study. Supplementary Figure 4. Vascular infiltration in patients with TAK. Supplementary Figure 5. Peripheral cytokine levels and their correlation with chemokine levels in patients with TAK. Supplementary Figure 6. Correlations between chemokines and disease activity markers in patients with TAK. Supplementary Figure 7. Correlations among different chemokines and changes in their levels after treatment. Supplementary Table 1. Clinical characteristics of the patients included for vascular tissue examination. Supplementary Table 2. Key functions of the five major chemokines. [file 13075_2022_2740_MOESM1_ESM.docx]

**Supplementary figure 1. Flow chart depicting the study design**

**
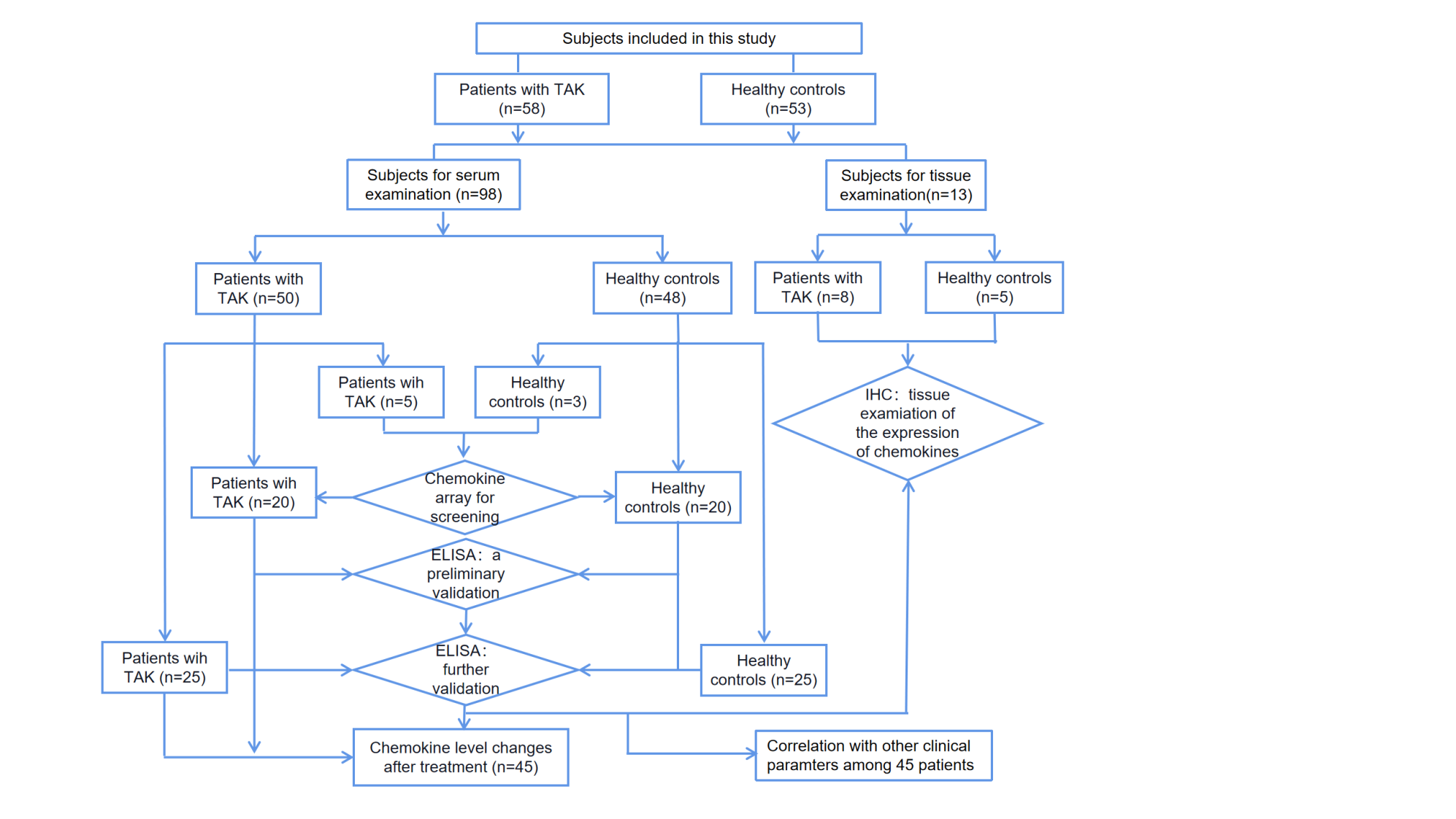
**

This study included 58 patients with TAK and 53 control subjects. Among them, 50 patients with TAK and 50 healthy controls were selected for serum examination, whereas 8 patients with TAK and 5 controls were selected for tissue examination. Among the participants selected for serum examination, 5 patients with TAK and 3 age- and sex-matched healthy controls were chosen for chemokine array detection. According to the array results, chemokines with 15% higher signals in patients with TAK were preliminarily validated in 20 patients with TAK and 20 healthy controls by ELISA. Then, the validated chemokines that were expressed at higher levels in patients with TAK were further detected in the remaining 25 patients and 25 healthy controls by ELISA. The results from these 25 patients and 25 controls were analyzed together to further confirm the elevated chemokines in patients with TAK. Following this, the expression of the final validated chemokines was detected in vascular tissue from patients with TAK and healthy controls by IHC to clarify their vascular expression. To clarify the effect of treatment on chemokine levels, the validated chemokines were also detected in 45 patients with TAK after treatment. Finally, the correlations between these chemokines and other clinical parameters were analyzed in the 45 patients with TAK.

**Supplementary figure 2. Gating strategies in flow cytometry for peripheral immune subsets analysis**

**
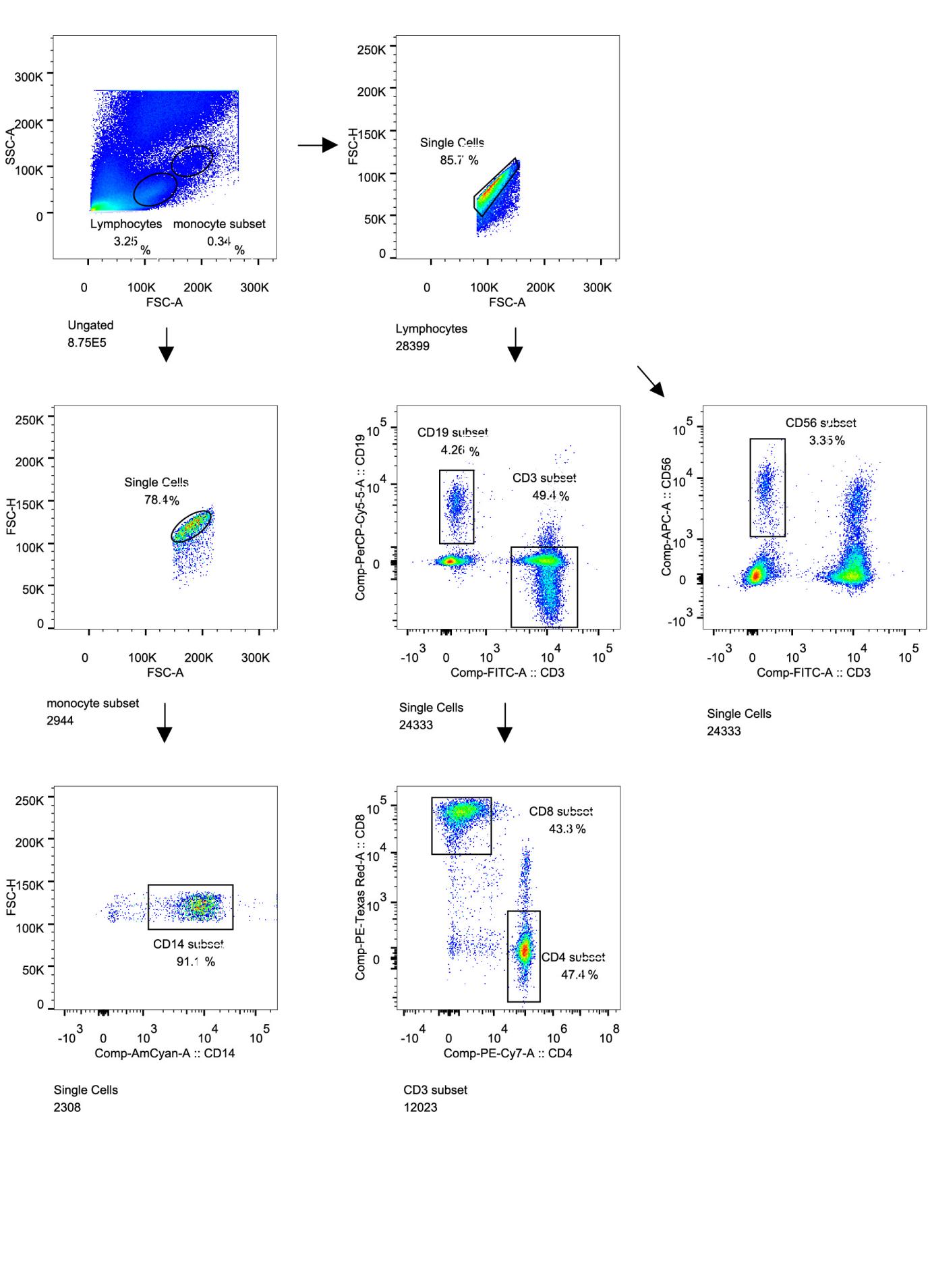
**

Lymphocytes and monocytes were gated firstly. From lymphocyte, CD3^+^ T cells were gated and then from it, CD4^+^ and CD8^+^ cells were gated to obtain CD3^+^CD4^+^ or CD3^+^CD8^+^ T cells. In addition, from lymphocytes, CD56^+^ NK cells were obtained by gating CD56^+^CD3^-^ cells. Similarly, CD19^+^ B cells were obtained by gating CD19^+^CD3^-^ cells. Monocyte was obtained by gating CD14^+^ cells.

**Supplementary figure 3. Results of chemokine array in the present study**

**
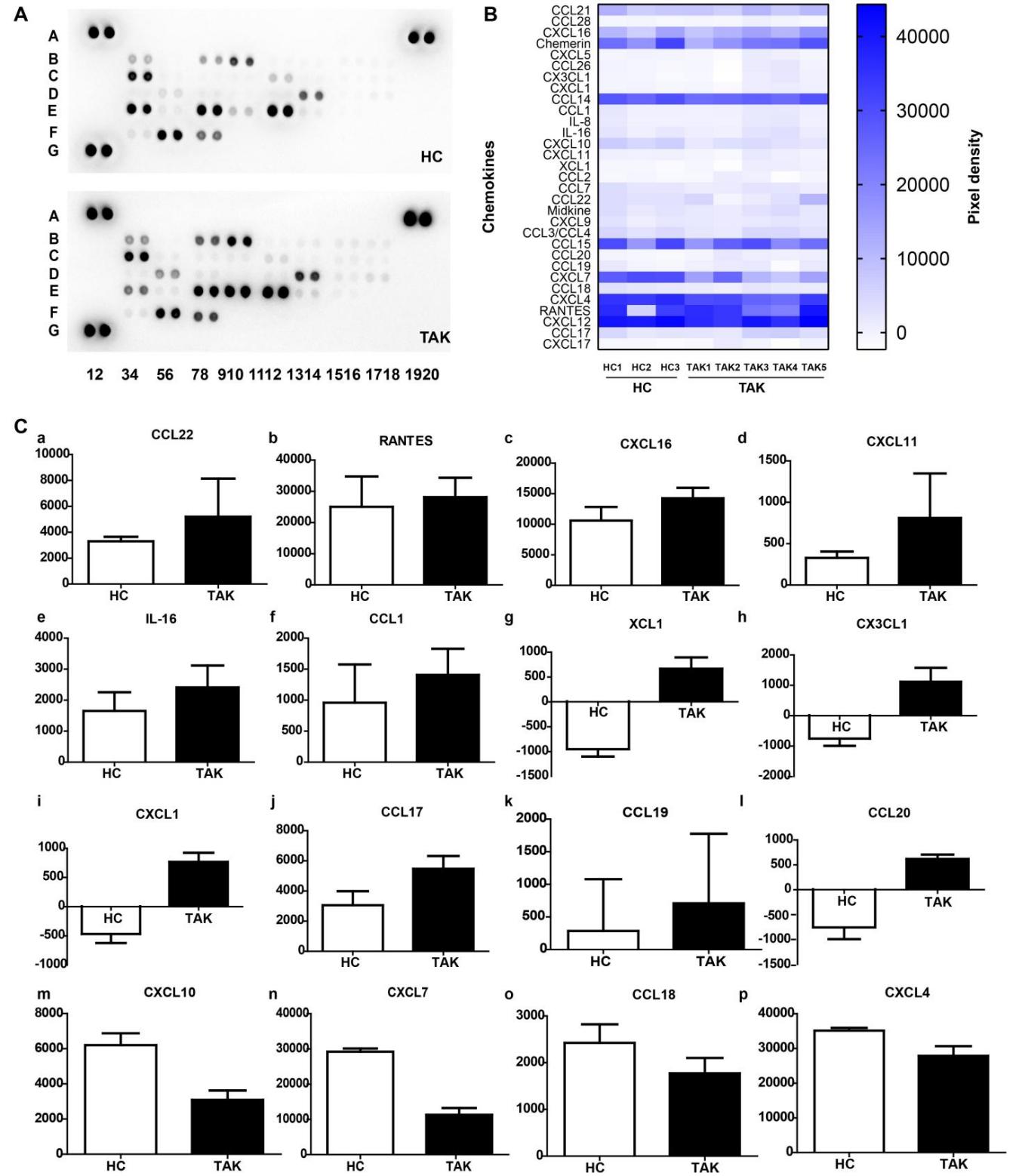
**

A: Representative image of the chemokine array assay performed in this study

A1, A2, A19, A20: reference spots

B3-B4: CCL21; B5-B6: CCL28; B7-B8: CXCL16; B9-B10: Chemerin; B11-B12: CXCL5; B13-B14: CCL26; B15-B16: CX3CL1; B17-B18: CXCL1; C3-C4: CCL14; C5-C6: CCL1; C7-C8: IL-8; C9-C10: IL-16; C11-C12: CXCL10; C13-C14: CXCL11; C15-C16: XCL1; C17-C18: CCL2; D3-D4: CCL7; D5-D6: CCL22; D7-D8: Midkine; D9-D10: CXCL9; D11-D12: CCL3/CCL4; D13-D14: CCL15; D15-D16: CCL20; D17-D18: CCL19; E3-E4: CXCL7; E5-E6: CCL18; E7-E8: CXCL4; E9-E10: RANTES; E11-E12: CXCL12; E13-E14: CCL17; E15-E16: CXCL17; F3-F4: Fibrinogen (control sample); F5-F6: gp130 (control sample); F7-F8: Transferrin R (control sample); F9-F10: negative control sample (-); G1-G2: reference spots

B: Heatmap results of the chemokine array assay in five patients with TAK and three healthy controls

C: Chemokines that had 15% higher signals in patients with TAK (CCL22, RANTES, CXCL16, CXCL11, IL-16, CCL1, XCL1, CX3CL1, CXCL1, CCL17, CCL19, and CCL20) (a–l) and 15% lower signals (CXCL10, CXCL, CCL18, and CXCL4) (m–p) in patients with TAK than in the healthy controls according to the chemokine array results.

HC: healthy control, TAK: Takayasu arteritis

**Supplementary figure 4. Vascular infiltration in patients with TAK**

**
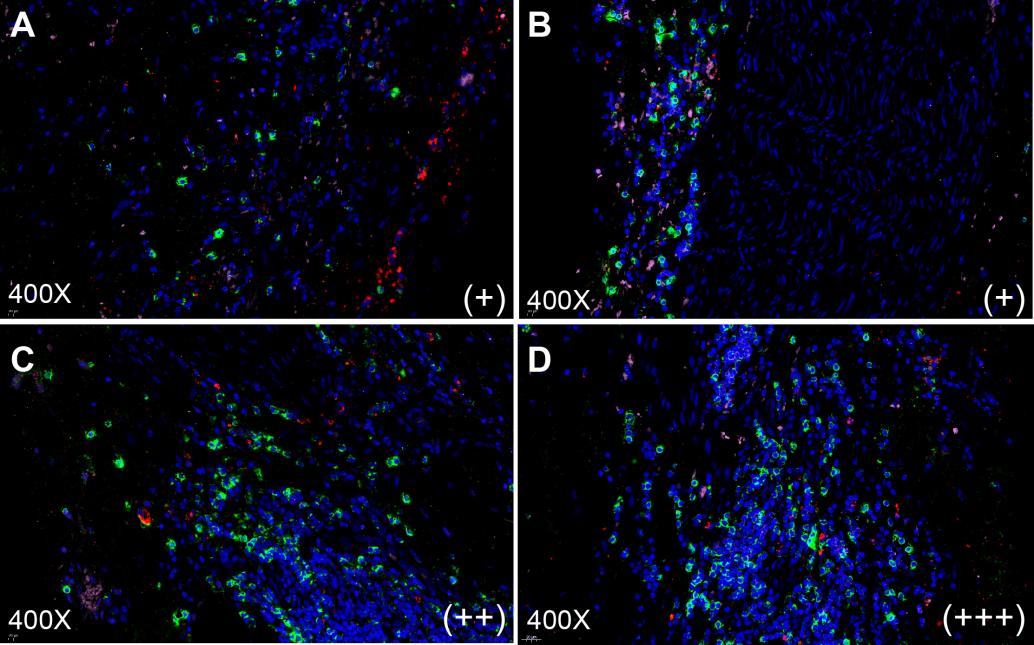
**

Different grades of vascular adventitial infiltration were shown in A (+), B (+), C(++), D(+++). Green (CD3), Red (CD68), Pink (CD19)

**Supplementary figure 5. Peripheral cytokine levels and their correlation with chemokine levels in patients with TAK**


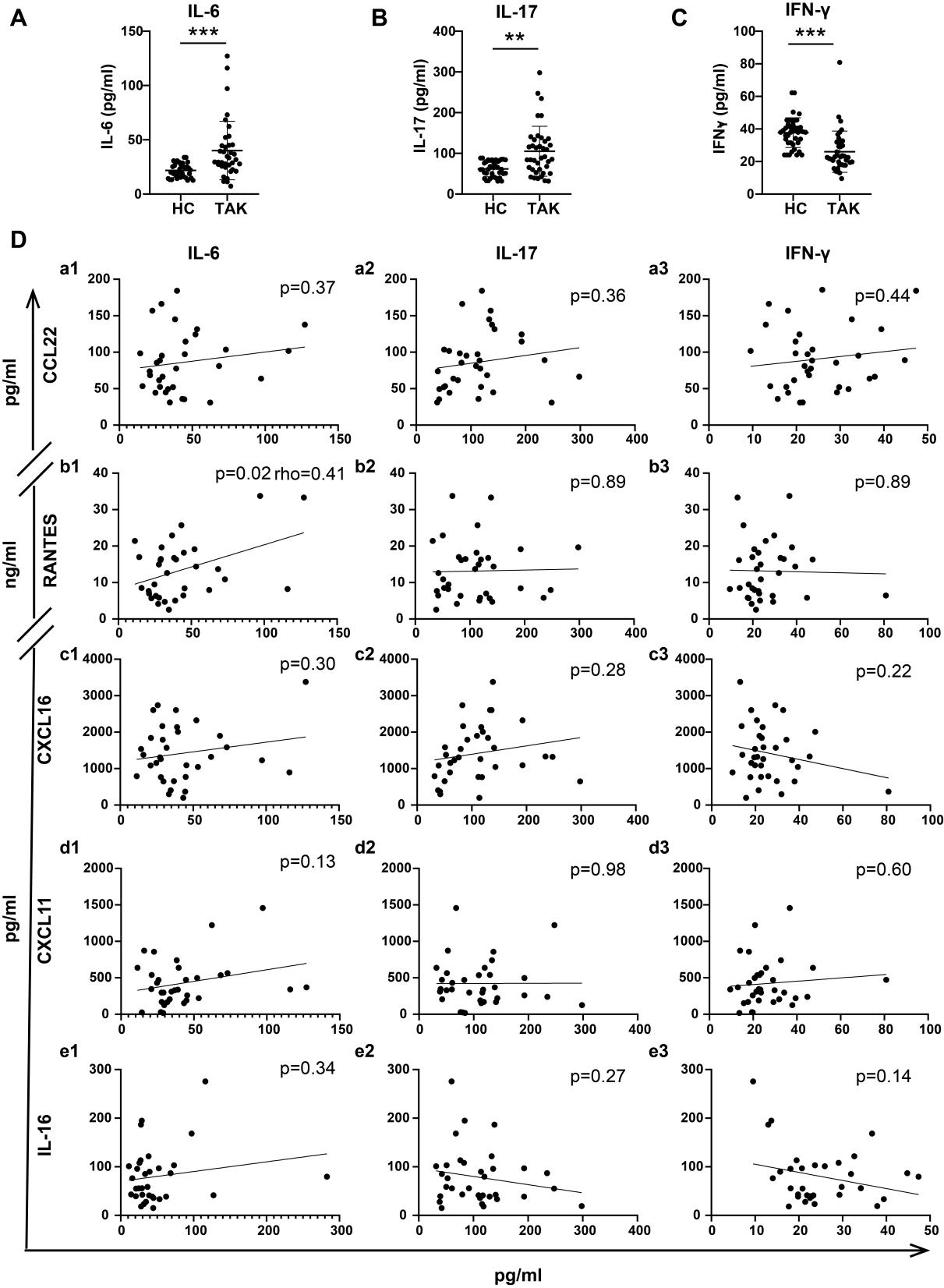


A–C: Levels of IL-6 (A), IL-17 (B), and IFN-γ (C) in patients with TAK and healthy controls (n = 45 in each group).

D: Correlations of CCL22 (a1–a3), RANTES (b1–b3), CXCL16 (c1–c3), CXCL11 (d1–d3), and IL-16 (e1–e3) levels with IL-6, IL-17, and IFN-γ levels.

**p < 0.01, ***p < 0.001

**Supplementary figure 6. Correlations between chemokines and disease activity markers in patients with TAK**


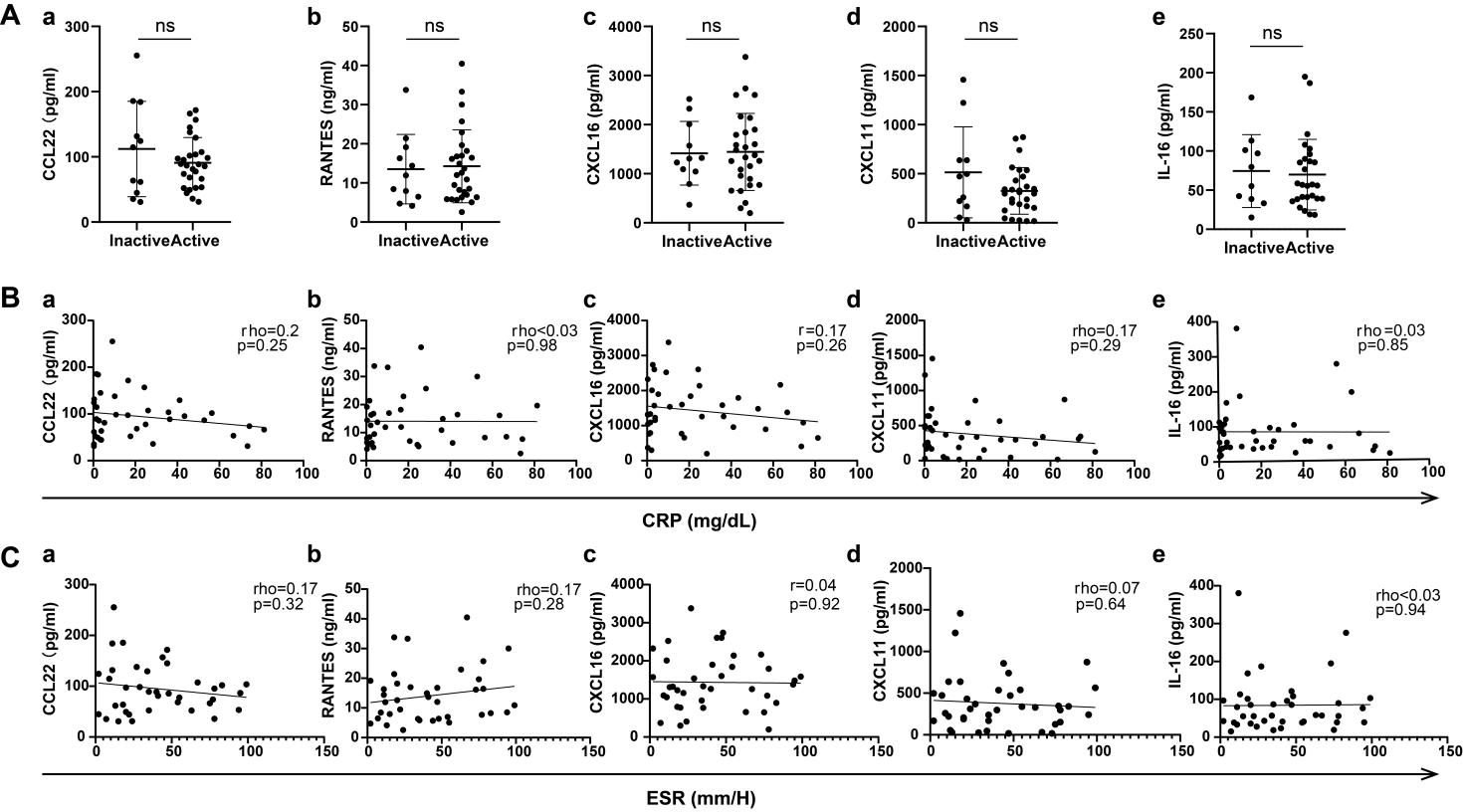


A: No differences were observed in CCL22 (a), RANTES (b), CXCL16 (c), CXCL11 (d), and IL-16 (e) levels between patients with inactive disease (n = 11) and those with active disease (n = 32).

B: No correlations were found between CRP levels and various chemokine levels, including CCL22 (a), RANTES (b), CXCL16 (c), CXCL11 (d), and IL-16 (e).

C: No correlations were found between ESR levels and various chemokine levels, including CCL22 (a), RANTES (b), CXCL16 (c), CXCL11 (d), and IL-16 (e).

**Supplementary figure 7. Correlations among different chemokines and changes in their levels after treatment**

**
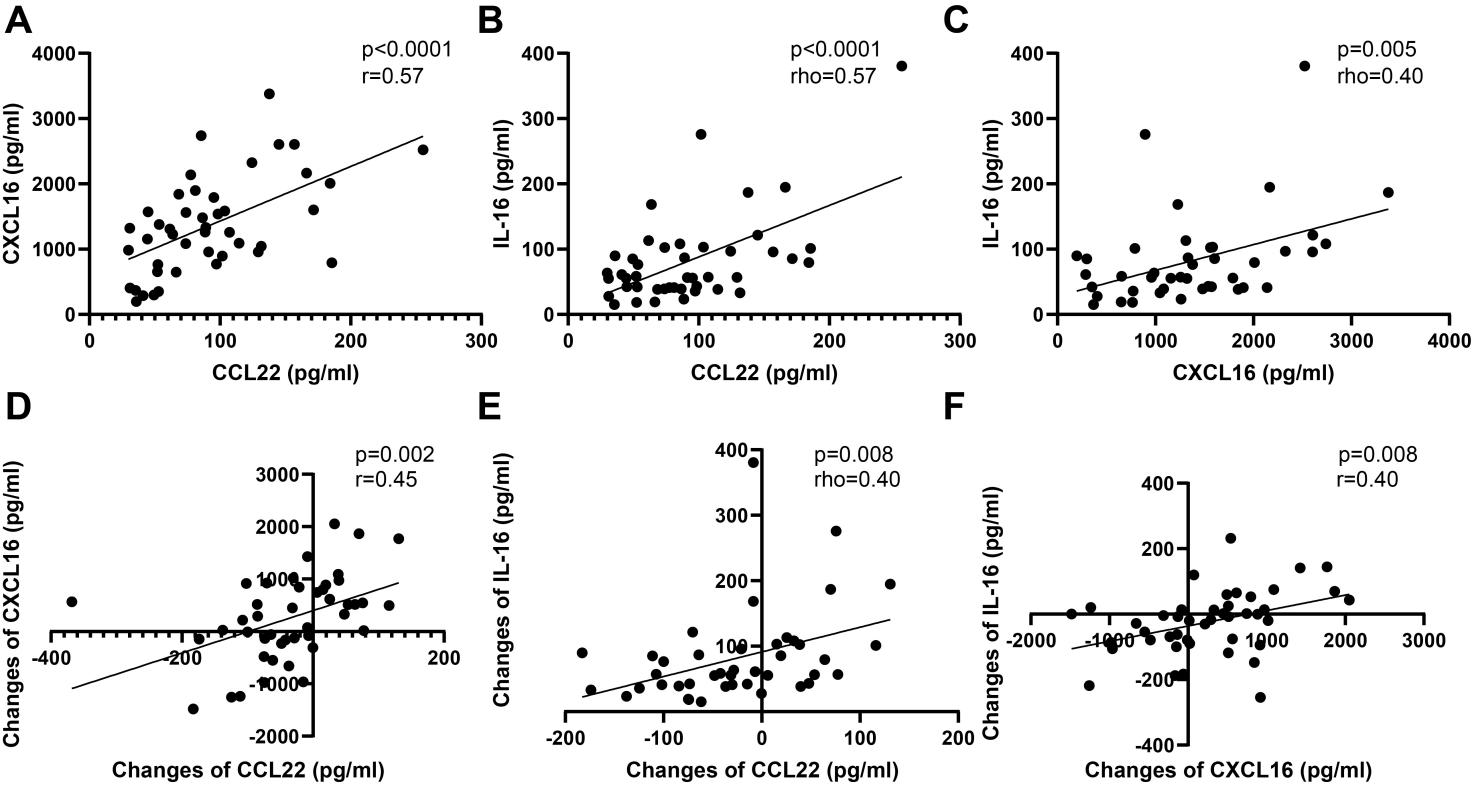
**

A: CCL22 was positively correlated with CXCL16 levels (r = 0.57, p < 0.001).

B: CCL22 was positively correlated with IL-16 levels (rho = 0.57, p < 0.001).

C: IL-16 was positively correlated with CXCL16 levels (rho = 0.40, p = 0.005).

D: Changes in CCL22 levels were positively correlated with changes in CXCL16 levels (r = 0.45, p = 0.002).

E: Changes in CCL22 levels were positively correlated with changes in IL-16 levels (rho = 0.40, p = 0.008).

F: Changes in IL-16 levels were positively correlated with changes in CXCL16 levels (r = 0.40, p = 0.008).

The changes were calculated as chemokine levels at the baseline minus the chemokine levels after treatment. A negative value indicated an increase in chemokine levels after treatment, while a positive value indicated a decrease after treatment.

**Supplementary Table 1. Clinical characteristics of the patients included for vascular tissue examination**

| **Subjects** | **TAK1** | **TAK2** | **TAK3** | **TAK4** | **TAK5** | **TAK6** | **TAK7** | **TAK8** | **Con1** | **Con2** | **Con3** | **Con4** | **Con5** |
| --- | --- | --- | --- | --- | --- | --- | --- | --- | --- | --- | --- | --- | --- |
| **Age at the time of surgery (y)** | 39 | 60 | 19 | 53 | 33 | 51 | 61 | 42 | 46 | 39 | 52 | 43 | 36 |
| **Gender** | M | F | F | F | M | F | F | M | F | M | M | F | F |
| **Specimen** | AA | AA | RA | AA | AA | AA | AV | AA | AA | AA | AbA | AbA | AA |
| **Reason for surgery** | AR | AR | RAS | AR | AR | AR | AR | AR | HT | HT | LT | LT | HT |
| **ESR (mm/H)** | / | 22 | 39 | 39 | 2 | 10 | 80 | 54 | / | / | / | / | / |
| **CRP (mg/L)** | 14.7 | 3 | 15.6 | 11.6 | 1.2 | 18.4 | 126.3 | 28.7 | / | / | / | / | / |
| **Imaging type** | I | I | IV | I | IIa | V | V | I | / | / | / | / | / |
| **Kerr score** | 3 | 3 | 3 | 3 | 2 | 2 | 3 | 3 | / | / | / | / | / |
| **Inflammation grade** | ++ | +++ | + | +++ | + | + | ++ | ++ | - | - | - | - | - |
| **CCL22** | ++ | ++ | + | ++ | + | + | ++ | ++ | - | - | - | - | - |
| **RANTES** | + | + | + | - | + | + | + | + | - | - | - | - | - |
| **CXCL16** | ++ | + | + | ++ | + | + | +++ | ++ | - | - | - | - | - |
| **CXCL11** | +++ | +++ | ++ | ++ | ++ | ++ | ++ | ++ | - | - | - | - | - |
| **IL-16** | +++ | ++ | ++ | +++ | ++ | ++ | ++ | ++ | - | - | - | - | - |

F: female; M: male; AA: ascending aorta; AbA: abdominal aorta; RA: renal artery; AV: aortic valve; AR: aortic regurgitation; RAS: renal artery stenosis; HT: heart transplantation; LT: liver transplantation; ESR: erythrocyte sedimentation rate; CRP: C-reactive protein; /: data missing or not applicable; Vascular inflammation: +++ (the average number > 100/0.15mm2), ++ (the average number 50-100/0.15mm2), + (the average number 10-50/0.15mm2), or － (the average number ＜10/0.15mm2); vascular expression: negative (－), weak positive (+), moderate positive (++) and strong positive (+++).

**Supplementary Table 2. Key functions of the five major chemokines**

| **Chemokine in the array** | **Alternative name** | **Receptor** | **Attraction cell types** |
| --- | --- | --- | --- |
| 6Ckine | CCL21 | CCR7, CCRL1 | NK, DCs, T cells |
| CCL28 | MEC | CCR3, CCR10 | Resting CD4 or CD8 T cells and eosinophils |
| **CXCL16** | **SR-PSOX** | **CXCR6** | **T cell, NK cell** |
| Chemerin | RARRES2 | ChemR23 | Immature plasmacytoid dendritic cells, immature myeloid DCs, macrophages and natural killer cells |
| ENA-78 | CXCL5 | CXCR2, DARC | Neutrophils |
| Eotaxin-3 | CCL26 | CCR3, CCR10 | Eosinophil |
| Fractalkine | CX3CL1 | CX3CR1 | NK, monocyte, macrophage, Th1 cell |
| GROalpha | CXCL1 | CXCR2, DARC | Neutrophils |
| HCC-1 | CCL14 | CCR1, CCR3, CCR5 | Monocytes, eosinophils, and T-cells |
| I-309 | CCL1 | CCR8 | Monocyte |
| IL-8 | CXCL8 | CXCR1, CXCR2, DARC | Neutrophils |
| **IL-16** | **LCF** | **CD4** | **CD4^+^ lymphocytes, monocytes, and eosinophils.** |
| IP-10 | CXCL10 | CXCR3 | Monocytes, natural killer and T cell |
| **I-TAC** | **CXCL11** | **CXCR3, CXCR7** | **Activated T cells** |
| Lymphotactin | XCL1 | XCR1 | Lymphocyte |
| MCP-1 | CCL2 | CCR2, CCR4 | Monocytes and basophils |
| MCP-3 | CCL7 | CCR1, CCR2, CCR3 | Monocytes and eosinophils |
| **MDC** | **CCL22** | **CCR4** | **Monocytes, dendritic cells, natural killer cells and for chronically activated T lymphocytes** |
| Midkine | NEGF2 | PTPRZ1, integrin | Smooth muscle cell, osteoblast cells, neuron |
| MIG | CXCL9 | CXCR3 | Lymphocyte |
| MIP-1alpha/beta | CCL3/CCL4 | CCR1, CCR4, CCR5 | T cell, monocyte |
| MIP-1delta | CCL15 | CCR1 | T cells monocyte |
| MIP-3alpha | CCL20 | CCR6 | B cell and DC |
| MIP-3beta | CCL19 | CCR7, CCRL1, CCRL2 | T cell and DC |
| NAP-2 | CXCL7 | CXCR2, DARC | Neutrophils |
| PARC | CCL18 | DARC | Naive T cells, CD4^+^ and CD8^+^ T cells, nonactivated lymphocytes |
| PF4 | CXCL4 | CXCR3 | Platelet |
| **RANTES** | **CCL5** | **CCR5** | **T cells, macrophages, NK cell, eosinophils, basophils** |
| SDF-1 | CXCL12 | CXCR4, CXCR7 | Neutrophils |
| TARC | CCL17 | CCR4, CCR8 | T lymphocytes |
| VCC-1 | CXCL17 | Unknown | Immature dendritic cells and blood monocytes |

SR-PSOX, scavenger receptor for phosphatidylserine and oxidized lipoprotein; RARRES2, retinoic acid receptor responder 2; NEGF2, Neurite Outgrowth-Promoting Factor 2; PTPRZ1, protein tyrosine phosphatase receptor type Z1; LCF, lymphocyte chemoattractant;

SDF-1, Stromal cell-derived factor 1; I-TAC, Interferon-inducible T-cell alpha chemoattractant; ENA-78, Epithelial Cell-derived Neutrophil-activating Peptide 78; MCP, Monocyte Chemoattractant Protein; MIP, Macrophage inflammatory protein; PF4, platelet factor 4; TARC, thymus and activation regulated chemokine; GRO-1, growth related oncogene peptide; VCC-1, Vascular endothelial growth factor-correlated chemokine 1; PARC, pulmonary and activation-regulated chemokine; NAP-2, neutrophil- activating peptide 2; MDC, Macrophage-derived chemokine; IP-10, human interferon-inducible protein 10; HCC-1, hemofiltrate CC chemokine-1; CCL, C-C motif ligand; CXCL, C-X-C Motif Chemokine Ligand; XCL, X-C Motif Chemokine Ligand

CCR,C-C motif chemokine receptor; CXCR, C-X-C motif chemokine receptor; XCR, X-C Motif Chemokine Receptor; DARC, Duffy Antigen Receptor for Chemokines; five chemokines significantly elevated in patients with TAK were highlighted.
